# Bayesian non-parametric inference for stochastic epidemic models using Gaussian Processes

**DOI:** 10.1093/biostatistics/kxw011

**Published:** 2016-03-18

**Authors:** Xiaoguang Xu, Theodore Kypraios, Philip D O'Neill

**Affiliations:** Institute of Population Health, University of Manchester, Manchester, UK; School of Mathematical Sciences, University of Nottingham, Nottingham, UK

**Keywords:** Bayesian non-parametrics, Epidemic model, Gaussian process, SIR model

## Abstract

This paper considers novel Bayesian non-parametric methods for stochastic epidemic models. Many standard modeling and data analysis methods use underlying assumptions (e.g. concerning the rate at which new cases of disease will occur) which are rarely challenged or tested in practice. To relax these assumptions, we develop a Bayesian non-parametric approach using Gaussian Processes, specifically to estimate the infection process. The methods are illustrated with both simulated and real data sets, the former illustrating that the methods can recover the true infection process quite well in practice, and the latter illustrating that the methods can be successfully applied in different settings.

## 1. Introduction

This paper is concerned with developing methods of Bayesian non-parametric inference for stochastic epidemic models which are partially observed through time. Our specific focus is on models of the susceptible-infective-removed (SIR) type in which only removals are observed. The following paragraphs contain the background and motivation for this work.

Methods for fitting stochastic models of disease transmission to outbreak data have been the subject of considerable research activity during the past fifteen years or so. Popular approaches include Markov chain Monte Carlo (MCMC) methods (Gibson and Renshaw, 1998; O'Neill and Roberts, 1999; O'Neill *and others*, 2000), Approximate Bayesian Computation methods (McKinley *and others*, 2009) and Sequential Monte Carlo methods (Ionides *and others*, 2006), all of which assume an underlying parametric model whose parameters are to be inferred from the data to hand. A natural alternative is to consider non-parametric approaches, which do not assume a specific parametric model and therefore offer greater potential flexibility. Another motivating factor in the context of epidemic models is that it is often unclear how best to assess the goodness of fit of parametric epidemic models, and hence hard to quantify the extent to which the underlying model assumptions are in line with observed data.

Non-parametric methods have to date received relatively little attention in the epidemic modeling literature, and to our knowledge there have been no previous Bayesian approaches. Both Becker and Yip (1989) and Becker (1989) consider non-parametric estimation of the infection rate in the so-called general epidemic model (i.e. the SIR model with infectious periods distributed according to an exponential distribution) by allowing the infection rate to depend on time. Their approach uses estimating equations, derived from suitable martingales, in combination with a suitable smoothing kernel. The approach also requires infection times to be known, which is rarely the case in reality, although by assuming fixed-length infectious periods the infection times are immediately specified by the observed removal times. In contrast, Lau and Yip (2008) assume only removals are observed, and use a kernel estimator to estimate the unobserved process of infectives, assuming that the parameter of the exponential infectious period distribution is known. Finally, Chen *and others* (2008) consider a related problem in which kernel estimation is used to estimate the infection rate in a large-scale epidemic model in which the depletion of susceptibles is ignored.

In this paper, we consider an SIR model in a closed population, and for simplicity and comparison with other methods, we assume exponentially distributed infectious periods. However, our methods can also be applied to general infectious period distributions. Throughout, we assume that the available data consist only of removal times, so that the infection process itself is unobserved. This assumption can usually be interpreted to mean that we observe case detection times, and that, at these times, the individuals in question are no longer able to infect others, perhaps due to isolation or other interventions. We assume that the infection mechanism is time-dependent, specifically making one of two assumptions.

First, we suppose that the usual overall incidence rate of new infections, $\beta X(t) Y(t)$, is replaced by $\beta (t) X(t) Y(t)$, where $X(t)$ and $Y(t$) are the numbers of susceptibles and infectives at time $t$, respectively. This approach is a natural starting point for non-parametric inference since it retains the usual natural mass-action assumption for new infections. In the second approach, we replace $\beta X(t) Y(t)$ by $\beta (t) \mathcal {I}_{\{ X(t)Y(t) \geq 1 \}}$, where $\mathcal {I}_A$ is the indicator function of the event $A$. A key motivation here is to relax the usual mass-action assumption, which at least informally enables us to investigate the validity of assuming a particular parametric form for the incidence rate.

Although the assumption that $\beta $ is constant over time is a common one in epidemic modeling, it is certainly not always realistic (Fang *and others*, 2004). Specifically, $\beta $ could vary over time as a result of factors such as behavior change in response to the epidemic, the introduction of control or mitigation measures, greater public awareness of the epidemic, and so on. There have been numerous approaches to estimating time-dependent infection rates for epidemic models, including the assumption of a particular parametric form with few parameters (Becker, 1989; Lekone and Finkenstadt, 2006) or many parameters (Ionides *and others*, 2006; Cauchemez and Ferguson, 2008), inference in deterministic models assuming that the number of infectives is known (Pollicott *and others*, 2012) and for modified deterministic models by assuming that the infection rate is a function of a diffusion process (Dureau *and others*, 2013). All of these approaches are distinct from that which we consider.

We adopt a Bayesian framework, and base our non-parametric modeling on Gaussian Processes (GPs). Specifically, we place GP prior distributions on the incidence rate or infection rate as appropriate, and adapt a method for estimating the intensity function of an inhomogeneous Poisson process (Adams *and others*, 2009) to our setting. The methods involve MCMC algorithms.

The paper is organized as follows. In Section 2, we define the epidemic models of interest, and briefly recall some key facts about GPs. In Sections 3 and 4, we present estimation methods for the infection rate and overall incidence rate, respectively. Section 5 contains some numerical illustrations of the methods, and we conclude in Section 6 with some discussion.

## 2. Background

### 2.1. Multitype SIR epidemic model

We first recall a multitype stochastic epidemic model (see, e.g. Becker and Hopper, 1983). In this model, individuals are grouped according to their susceptibility to disease, but all individuals are equally infectious if they become infected.

Consider a population consisting of $k$ groups, labeled $1, \ldots , k$, such that the $i$th group initially contains $N_i$ susceptible individuals. At any time, each individual in the population can be in one of three states, namely susceptible, infective, or removed. An epidemic is initiated in this population at time $t=0$ by one of the susceptibles becoming infective. For $t \geq 0$ and $i = 1, \ldots , k$ denote by $X_i(t)$ and $Y_i(t)$, respectively, the numbers of susceptibles and infectives in the $i$th group at time $t$. Let $Y(t) = \sum _{i=1}^k Y_i(t)$ denote the total number of infectives in the population at time $t$. The epidemic can be defined as a bivariate Markov chain with the following transition probabilities, which correspond, respectively, to an infection in group $i$ and a removal:

\begin{align*} {\rm{Pr}} ((X_i(t+h), Y(t+h)) = (x-1,y+1)\, |\, (X_i(t),Y(t))=(x,y)) &= \beta_i xy h + o(h),\\ {\rm{Pr}} ((X_i(t+h), Y(t+h)) = (x,y-1) \,|\, (X_i(t),Y(t))=(x,y)) &= \gamma y h + o(h), \end{align*}

with all other transitions having probability $o(h)$. It follows that (i) the overall rate of new infections, which we refer to as the incidence rate, is given by $\sum _{i=1}^k \beta _i X_i(t) Y(t)$, and (ii) individuals have infectious periods that are independently distributed according to an exponential distribution with mean $\gamma ^{-1}$. The $\beta _i$ parameters are called infection rates. If $k=1,$ there is only one group, the model reduces to the general stochastic epidemic model (see, e.g. Bailey, 1975), and in this case we write $\beta $ for $\beta _1$.

### 2.2. GPs and inference for Poisson processes

We now briefly review relevant facts about GPs (see, e.g. Rasmussen and Williams, 2006), and a method of Bayesian non-parametric inference for time-inhomogeneous Poisson processes that we shall adapt to our setting. Recall that a GP is a stochastic process whose realizations consist of Gaussian random variables indexed by some set (in our case, the set of times $t$ in some interval). A GP is completely specified by its mean and covariance function. Throughout this paper, we will only use zero-mean GPs, so that we need only specify a covariance function. GPs are commonly used in Bayesian inference to act as prior distributions over spaces of functions which are themselves the object of inference. We will use GPs as prior distributions for infection rate and incidence rate functions.

A closely related problem to that we will consider is that of Bayesian non-parametric inference for a time-inhomogeneous Poisson process. Specifically, suppose we observe a set of points $\{s_k\}^{K}_{k=1}$ from a Poisson process with time-dependent intensity $\lambda (t)$ during $[0,T]$. The likelihood is specified by

\[ \pi( \{s_k\}^{K}_{k=1} \,|\, \lambda) = \prod_{k=1}^K \lambda(s_k-) \exp \left\{ - \int_0^T \lambda(t) \, \mathrm{d}t \right\} , \]

where $\lambda (s_k-) = \lim _{t \uparrow s_k} \lambda (t)$. If $\lambda $ is the object of inference, then in the non-parametric setting, $\lambda $ is an infinite-dimensional object (specifically, it is defined by the set of values $\{ \lambda (t): 0 \leq t \leq T \}$), which in turn makes the integral in the likelihood intractable in practice.

A method for overcoming this difficulty is described in Adams *and others* (2009). The key idea is that the original process can be viewed as a thinned homogeneous Poisson process of rate $\lambda ^*$, where $\lambda (t) \leq \lambda ^*$ for all $0 \leq t \leq T$, in which each point is retained with probability $\lambda (t)/\lambda ^*$ (see Lewis and Shedler, 1979). Inference then proceeds by augmenting the observed data with the unobserved thinned points, $\{\tilde {s}_m\}^{M}_{m=1}$, say, yielding an augmented likelihood

\[ \pi( \{s_k\}^{K}_{k=1}, M, \{\tilde{s}_m\}^{M}_{m=1}\,|\, \lambda, \lambda^*) = (\lambda^{*})^{M+K}\exp\{-\lambda^{*}T\} \prod_{k=1}^K \frac{\lambda(s_k-)}{\lambda^*} \prod_{m=1}^M \left( 1 - \frac{\lambda(\tilde{s}_m-)}{\lambda^*} \right) . \]

Finally, since $\lambda \geq 0,$ it cannot be assigned a GP prior distribution directly, but instead we use the transformation $\lambda (t) = {\lambda }^{*}{\sigma }(g(t))$, where ${\sigma }(z)=(1+e^{-z})^{-1}$, and assign a GP prior distribution to $g$. This prior distribution is specified by assuming a particular form of covariance function with parameter vector $\theta $, and placing a prior distribution on $\theta $.

Let $\textbf {g}_{M+K} = (g(s_{1^-}),g(s_{2^-}),\ldots ,g(s_{K^-}), g(\tilde {s}_{1^-}),g(\tilde {s}_{2^-}),\ldots ,g(\tilde {s}_{M^-}))$. Then from Bayes' Theorem the posterior density of interest is

\begin{align*} \pi(g, \lambda^*, \{\tilde{s}_m\}^{M}_{m=1}, M, \theta \,|\, \{s_k\}^{K}_{k=1} ) & \propto (\lambda^{*})^{M+K}\exp\{-\lambda^{*}T\}\prod_{k=1}^{K}\sigma(g(s_{k}-))\prod_{m=1}^{M}\sigma(-g(\tilde{s}_m-))\\ &\quad \times {\pi(\textbf{g}_{M+K}|\{s_k\}^{K}_{k=1},\{\tilde{s}_m\}^{M}_{m=1},\theta)} \pi(\lambda^*) \pi (\theta), \end{align*}

where $\pi (\textbf {g}_{M+K}\,|\,\{s_k\}^{K}_{k=1},\{\tilde {s}_m\}^{M}_{m=1},\theta )$ is the density of a multivariate Gaussian random variable, and $\pi (\lambda ^*)$ and $\pi (\theta )$, respectively, are the prior density functions of $\lambda ^*$ and $\theta $. In practice these prior distributions are often fairly uninformative; note also that a Gamma prior distribution for $\lambda ^*$ is (conditionally on the data and other parameters) conjugate. The posterior density itself can be explored using MCMC methods as described in Adams *and others* (2009), and since $\lambda $ is specified by $g$ and $\lambda ^*$, we can hence obtain posterior samples for $\lambda $.

## 3. Non-parametric inference for the infection rate

In this section, we modify the multitype SIR model by replacing the group-$i$ infection rate $\beta _i$ with $\tilde {\beta }_i(t)$, and then describe how to estimate the latter in a Bayesian non-parametric framework. Thus the transition probabilities governing the model become

\begin{align*} {\rm{Pr}} ((X_i(t+h), Y(t+h)) = (x-1,y+1)\,|\, (X_i(t),Y(t))=(x,y)) & = \tilde{\beta}_{i}(t) xy h + o(h),\\ {\rm{Pr}} ((X_i(t+h), Y(t+h)) = (x,y-1)\, |\, (X_i(t),Y(t))=(x,y)) & = \gamma y h + o(h). \end{align*}

We assume that the data consist of the unique group membership of each individual in the population, the removal times in each group, and the initial numbers of susceptibles $N_1, \ldots , N_k$. For convenience, we assume time zero is the time of the first observed removal time and that all removals are observed during $[0,T]$. Our objective is to infer both the infection rate functions $\tilde {\beta }_1, \ldots , \tilde {\beta }_k$ and the removal rate $\gamma $. We start with some notation.

For $i=1, \ldots , k$, let $n_i$ and $m_i$ denote, respectively, the numbers of observed removals and unobserved infections in group $i$. Let ${{\boldsymbol R}}_i=(R_{i1},R_{i2},\ldots ,R_{in_i})$ denote the set of ordered removal times in group $i$, so that $R_{i1} \leq R_{i2} \leq \cdots \leq R_{in_i}$, and let $\textbf {I}_i=(I_{i1},I_{i2},\ldots ,I_{im_i})$ denote the vector of ordered infection times in group $i$. Set $I_{\text {min}}$ as the time of the initial infection and $i_{\text {min}}$ as the group of the initial infective, so that if $j = i_{\text {min}}$, then $I_{j1} = I_{\text {min}}$. Let $\textbf {I}=(\textbf {I}_1,\textbf {I}_2,\ldots ,\textbf {I}_k) \setminus I_{\text {min}}$ denote all infection times other than the initial infection time. Finally, set ${{\boldsymbol R}}=({{\boldsymbol R}}_1,{{\boldsymbol R}}_2,\ldots ,{{\boldsymbol R}}_k)$, $\tilde {\boldsymbol {\beta }}(t)=(\tilde {\beta }_1(t),\tilde {\beta }_2(t),\ldots ,\tilde {\beta }_k(t))$, $\textbf {N}=({N}_1,{N}_2,\ldots ,{N}_k)$ and let $N=\sum _{i=1}^{k}{N_i}$ denote the initial number of susceptibles in the whole population.

The likelihood of the observed data ${{\boldsymbol R}}$ given the model parameters is analytically and computationally intractable in all but the simplest cases, essentially because its calculation requires integrating over all possible unobserved infection events. We therefore adopt the data augmentation approach in Hayakawa *and others* (2003) in which the unobserved infection times become model parameters. This leads to the augmented likelihood

\begin{align*} \pi(\textbf{I},{{\boldsymbol R}}|\tilde{\boldsymbol{\beta}}(t),\gamma,I_{\text{min}}) & = \left(\prod_{i=1}^{k}\left(\prod_{j=1}^{n_i}{\gamma}Y_i(R_{ij^-})\right) \left(\prod_{l=2}^{m_i}\tilde{\beta}_i(I_{il^-})X_i(I_{il^-})Y(I_{il^-})\right)\right) \\ &\quad \times \left(\prod_{i=1,i\neq{i_{{\rm min}}}}^{k}\tilde{\beta}_i(I_{i1^-})X_i(I_{i1^-})Y(I_{i1^-})\right)\\ &\quad \times\exp\left(-\sum_{i=1}^{k}\left({\int}_{I_{{\rm min}}}^{T}\tilde{\beta}_i(s)X_i(s)Y(s)\,\mathrm{d}s+{\int}_{I_{i1}}^{T}{\gamma}Y_i(s)\,\mathrm{d}s\right)\right). \end{align*}

We now place a prior distribution on $\tilde {\beta }_i$ by setting ${\tilde {\beta }_i}(t)={\tilde {\beta }_i}^{*}{\sigma }(g_i(t))$, where ${\tilde {\beta }_i}^{*}$ is an upper bound on $\tilde {\beta }_i(t)$, ${\sigma }(z)=(1+e^{-z})^{-1}$ and $g_i$ has a GP distribution with parameter vector $\theta _i$. As for the Poisson process example described above, this makes the augmented likelihood intractable, so we proceed by regarding the infection process as a thinned homogeneous Poisson process, as follows.

For group $i$ for which $I_{i1}=I_{{\rm min}}$, we introduce the additional variables (i) the number of thinned events, ${M}_i$; (ii) the locations of the thinned events, $\tilde {\textbf {I}}_i= (\tilde {I}_{i1},\ldots ,\tilde {I}_{iM_{i}})$; (iii) the $g_i$ function values at the infection times, $\textbf {g}_{m_i}=(g_i(I_{i2^-}),\ldots ,g_i(I_{im_i^-}));$ and (iv) the $g_i$ function values at the locations of thinned events, $\textbf {g}_{M_i}=(g_i(\tilde {I}_{i1^-}),\ldots ,g_i (\tilde {I}_{iM_i^-}))$. For groups $i$ with $I_{i1}\neq {I_{\text {min}}}$, we introduce the corresponding variables but also need to consider the first infection time, $I_{i1}$, in group $i$. Specifically, $g_i(I_{i1^-})$ is added to $\textbf {g}_{m_i}$. The augmented likelihood thus becomes

\begin{align*} &\pi(\textbf{I},{{\boldsymbol R}},\{M_i,\tilde{\textbf{I}}_i,\textbf{g}_{M_i+m_i}\}_{i=1}^{k}|\{\tilde{\beta}_i^*, \theta_i \}_{i=1}^{k},\gamma,I_{\text{min}})\\ &\quad =\left(\prod_{i=1}^{k}\left(\prod_{j=1}^{n_i}{\gamma}Y_i(R_{ij^-})\right)\left(\prod_{l=2}^{m_i}\tilde{\beta}_i^*X_i(I_{il^-})Y(I_{il^-})\sigma(g_i(I_{il^-}))\right)\right)\\ &\qquad \times\left(\prod_{i=1}^{k}\left(\prod_{s=1}^{M_i}\tilde{\beta}_i^{*}X_i(\tilde{I}_{is^-}) Y(\tilde{I}_{is^-})\sigma(-g_i(\tilde{I}_{is^-}))\right)\right) \left(\prod_{i=1,i\neq{i_{\text{min}}}}^{k}\tilde{\beta}_i^*X_i(I_{i1^-})Y(I_{i1^-})\sigma(g_i(I_{i1^-}))\right)\\ &\qquad \times\exp\left(-\sum_{i=1}^{k}\left({\int}_{I_{\text{min}}}^{T}\tilde{\beta}_i^*X_i(s)Y(s)\,\mathrm{d}s+{\int}_{I_{i1}}^{T}{\gamma}Y_i(s)\,\mathrm{d}s\right)\right)\\ &\qquad \times\left(\prod_{i=1}^{k}\pi(\textbf{g}_{M_i+m_i}|M_i,\{I_{il}\}^{m_i}_{l=1+\delta_i},\{\tilde{I}_{is}\}^{M_i}_{s=1},\theta_i)\right), \end{align*}

where $\textbf {g}_{M_i+m_i}$ is the concatenation of $\textbf {g}_{M_i}$ and $\textbf {g}_{m_i}$ and $\delta _i= \mathcal {I}_{\{i=i_{\text {min}} \} }$. Note that the values of $X_i(t)$ and $Y_i(t)$ do not change at the times of the thinned events. The posterior density of interest is then given by

\begin{align*} &\pi(\textbf{I},\{M_i,\tilde{\textbf{I}}_i,\textbf{g}_{M_i+m_i}\}_{i=1}^{k}, \{\tilde{\beta}_i^*, \theta_i \}_{i=1}^{k},\gamma,I_{\text{min}}\, |\, {{\boldsymbol R}}) \\ &\quad \propto \pi(\textbf{I},{{\boldsymbol R}},\{M_i,\tilde{\textbf{I}}_i,\textbf{g}_{M_i+m_i}\}_{i=1}^{k}|\{\tilde{\beta}_i^*, \theta_i \}_{i=1}^{k},\gamma,I_{\text{min}}) \pi (\{\tilde{\beta}_i^* \}) \pi (\gamma) \pi (I_{\text{min}}) \pi (\{\theta_i\}), \end{align*}

where the final four terms denote prior densities. Note that we assume independent prior distributions; this can of course be relaxed, but independence seems the most natural assumption. Samples from the posterior density can be obtained using MCMC methods, as described in the online supplementary material (available at *Biostatistics* online).

## 4. Non-parametric inference for the overall incidence rate

We now adapt the methods described above to the situation in which the mass-action assumption is relaxed. Specifically, we are interested in estimating the overall incidence rate in the absence of any particular parametric form for the infection process model. We restrict attention to the single-type epidemic, in keeping with the motivation to make as few assumptions as possible about the infection mechanism. However, the methods could be extended to multitype models.

The model of interest is a single-type SIR model in which the overall incidence rate $\beta X(t)Y(t)$ is replaced by $\beta (t) \mathcal {I}_{ \{ X(t), Y(t) \geq 1 \}}$, where $\mathcal {I}_A$ denotes the indicator function of the event $A$. Thus infections can only occur if there is at least one infective and one susceptible. The epidemic can be described according to the transition probabilities

\begin{align*} {\rm{Pr}} ((X(t+h), Y(t+h)) = (x-1,y+1)\,|\, (X(t),Y(t))=(x,y)) & = \beta(t)\mathcal{I}_{\{x, y \geq 1\}} h + o(h),\\ {\rm{Pr}} ((X(t+h), Y(t+h)) = (x,y-1)\,|\, (X(t),Y(t))=(x,y)) & = \gamma y h + o(h). \end{align*}

As before, we assume that we observe only the removal times and the initial number of susceptibles. Our objective is to infer both $\beta (t)$ and $\gamma $.

Denote the observed ordered removal times in $[0,T]$ as ${\boldsymbol R}=(R_1=0,R_2,\ldots ,R_K)$, so $R_1\leq R_2 \leq \cdots \leq R_K \leq T$. Let $I_1 < 0$ be the unobserved time of the first infection and let $\textbf {I}=(I_2,I_3,\ldots ,I_K)$ denote the remaining unobserved ordered infection times during $[I_1,T]$, so $I_1\leq {I_2}\leq \cdots \leq {I_K}$. We assume that the epidemic is known to have ceased by time $T$, i.e. the number of infection times and removal times are equal. In order that the epidemic does not die out before all the observed removals have occurred, we require that $I_{i} \leq I_{i+1} \leq R_i$ for $i=2,3,\ldots ,K-1$. The augmented likelihood of the removal times and unobserved infection times is

\[ \pi({\boldsymbol R}, \textbf{I}|\beta(t), \gamma, I_1) = \chi \prod_{j=2}^{K}\beta(I_j-) \exp\left\{-\int_{I_1}^{T}\beta(s)\,\mathrm{d}s\right\} \prod_{i=1}^{K}{\gamma}Y({R_i-}) \exp\left\{-\int_{I_1}^{T}{\gamma}Y({s})\,\mathrm{d}s\right\}; \]

see, for example, O'Neill and Roberts (1999), where $\chi $ denotes the indicator function of the event that $X(I_j-), Y(I_j-) \geq 1$ for $j=2, \ldots , K$, i.e. $\chi = 1$ if and only if there is at least one infective and one susceptible present when each infection event occurs.

As before, we place a GP prior on $\beta $ by setting $\beta (t)=\beta ^*{\sigma }(g(t))$, where $\beta ^{*}$ is an upper bound on $\beta (t)$, ${\sigma }(z)=(1+e^{-z})^{-1}$, and $g$ is a random function that has a GP distribution with parameter vector $\theta $. Suppose that the (unobserved) set of thinned infection events is $\{\tilde {I}_s\}^{M}_{s=1}$ and define $\textbf {g}_{M}=(g(\tilde {I}_{1^-}), g(\tilde {I}_{2^-}),\ldots ,g(\tilde {I}_{M^-}))$ as the vector of values of $g$ at these events. Similarly define $\textbf {g}_{K}=(g(I_{2^-}),g(I_{3^-}),\ldots ,g(I_{K^-}))$ as the vector of values of $g$ at the infection times, and denote by $\textbf {g}_{M+K}$ the concatenation of $\textbf {g}_{M}$ and $\textbf {g}_{K}$. We then obtain the augmented likelihood

\begin{align*} &\pi({\boldsymbol R},\textbf{I},M,\{\tilde{I}_s\}^{M}_{s=1},\textbf{g}_{M+K}\,|\,\beta^*,\gamma,I_1,\theta) \propto \chi (\beta^{*})^{K+M-1}\exp(-\beta^*(T-I_1)) \\ &\quad \times \prod_{j=2}^{K}\sigma\big(g(I_{j^{-}})\big)\prod_{s=1}^{M}\sigma\big(-g(\tilde{I}_{s^{-}})\big) \prod_{i=1}^{K}{\gamma}Y(R_{i^{-}})\exp\left\{-{\int}_{I_1}^{T}{\gamma}Y(s)\,\mathrm{d}s\right\} \pi\big(\textbf{g}_{M+K}\,|\,M,\textbf{I},\{\tilde{I}_s\}^{M}_{s=1},\theta\big), \end{align*}

and the posterior density of interest is

\begin{align*} &\pi (\textbf{I},M,\{\tilde{I}_s\}^{M}_{s=1},\textbf{g}_{M+K}, \beta^*, \gamma, I_1, \theta \,|\,{\boldsymbol R})\\ &\quad \propto \pi({\boldsymbol R},\textbf{I},M,\{\tilde{I}_s\}^{M}_{s=1},\textbf{g}_{M+K}\,|\,\beta^*,\gamma,I_1,\theta) \pi(\beta^*) \pi (\gamma) \pi(I_1) \pi (\theta), \end{align*}

where $\pi (\beta ^*)$, $\pi (\gamma )$, $\pi (I_1),$ and $\pi (\theta )$ denote prior densities. The posterior density can be explored using MCMC methods as described in the online supplementary material (available at *Biostatistics* online).

## 5. Examples

We now illustrate our methods via some examples. Simulated data are used to show that the methods work in practice. We then consider two classical data sets in small populations, and finish with a more elaborate example to show that the methods can also be extended to larger population settings.

Unless stated otherwise, we use the squared exponential covariance function for the GP prior (see, e.g. Rasmussen and Williams, 2006), i.e.

\[ K(x,y) = \alpha^2 \exp [ - (x-y)^2 / (2 \theta^2)], \]

with $\alpha = 2$. We comment on the use of different priors later. The prior distribution for the hyperparameter $\theta $ was set to be $\mbox {Exp}(0.1)$, where $\mbox {Exp}(\lambda )$ denotes an exponential random variable with mean $\lambda ^{-1}$. The parameters $\gamma $, $\tilde {\beta }^*_i$, and $\beta ^*$ were assigned independent $\mbox {Exp}(0.0001)$ prior distributions, which, for the examples below corresponds to fairly uninformative prior beliefs. In general, the choice of prior distribution will be problem-specific, depending on the time scale in question. Finally, we defined a time axis by setting the first removal time equal to time zero and then set $-I_{\text {min}} \sim \mbox {Exp}(0.1)$ a priori.

### 5.1. Simulated data

Full details of a simulation study can be found in the online supplementary material (available at *Biostatistics* online). We considered three scenarios, namely a single-type model with constant infection rate, a single-type model with infection rate exponentially decreasing through time, and a multitype model with three types of individual. In each case we simulated 50 data sets, and applied our methods to each resulting data set. In each scenario, we found that the true infection rate was well estimated on average across the simulations, while estimates based on single data sets were also reasonable, even for relatively small epidemic outbreaks.

### 5.2. Abakaliki smallpox data

We now consider a classic smallpox data set taken from Bailey (1975, p. 125). The data were originally reported in a World Health Organization report (Thompson and Foege, 1968) and the time series of 30 case detection times, assuming a homogeneously mixing population of 120 individuals, have since been analyzed by numerous authors (e.g. Bailey and Thomas, 1971; Becker, 1989; Rida, 1991; O'Neill and Roberts, 1999; Boys and Giles, 2007 and references therein), while Eichner and Dietz (2003) provide a far more comprehensive analysis that takes into account the mixing structure of the population and other important factors. In terms of non-parametric analyses, Becker and Yip (1989) assume known infection times and latent periods, and use a kernel smoothing method to estimate the infection rate as a function of time, concluding that the infection rate displays some oscillation over time but with a gradual downward trend during the outbreak. Finally, Lau and Yip (2008) use kernel smoothing to obtain a non-parametric estimate of the trajectory of infectives, but assume the infection rate itself is fixed.

**Fig. 1.**
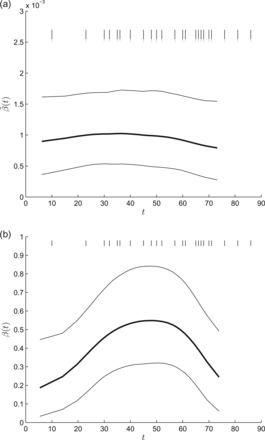
Estimation of the infection rate (a) and incidence rate (b) for the Abakaliki smallpox data. Both plots show the posterior mean (thick line), 95% posterior credible intervals (thin line), and the days on which cases were detected in the data set (vertical dashes at top of plot). All curves are plotted over the mean posterior time during which infectives were present in the population.

In our analysis, we assume that only case detection times are available, corresponding to removal times. The infection times for each individual and the removal rate are all inferred via the MCMC algorithm. Figure 1 shows posterior summaries for the infection rate and incidence rate. The infection rate shows a slight initial increase followed by a slight gradual decline. The fact that we do not see the oscillations in the Becker–Yip analysis is most likely due to the fact that our estimation of the infection times provides a degree of smoothing. The incidence rate curve peaks at around $t=50$, which is around the time that control measures were estimated to have been introduced during the outbreak (Eichner and Dietz, 2003). For comparison, if the standard SIR model is fitted in a Bayesian framework, the posterior mean (standard deviation) of the infection rate $\beta $ is $0.94 \times 10^{-3}$ ($0.19 \times 10^{-3}$) (Fearnhead and Meligkotsidou, 2004), which is evidently very similar to the values taken by $\tilde {\beta }(t)$ in our analysis.

### 5.3. Tristan da Cunha respiratory disease data

We also apply our methods to a data set analyzed in Becker and Hopper (1983) and Hayakawa *and others* (2003). The data set corresponds to removal times of individuals with a respiratory disease which occurred between October and November of 1967 on the island of Tristan da Cunha in the South Atlantic. The total population of the island of 255 was partitioned into three groups by age: infants, children, and adults. As there was one unidentified case, we suppose that $N=254$. The initial number of susceptibles are $N_1=25$, $N_2=36,$ and $N_3=192$, and we assume that the initial infective is an adult since the first case was an adult and occurred 9 days before the first non-adult case. The number of cases in each group was 9 (infants), 6 (children), and 25 (adults).

**Fig. 2.**
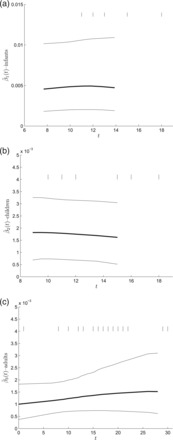
Estimation of the infection rates for infants, children, and adults for the Tristan da Cunha respiratory disease data. All plots show the posterior mean (thick line), 95% posterior credible intervals (thin line), and the days on which cases were detected in the data set (vertical dashes at top of plot). All curves are plotted over the mean posterior time during which infectives were present in the population.

Figure 2 shows the estimation results for the infection rate of each group. The infection rate among children falls slightly during the outbreak whilst that for adults gradually increases. These findings are not unreasonable given the pattern of removals in these groups; for instance, most of the adult cases occur in the second half of the outbreak. Nevertheless, it is also clear that assuming constant infection rates would not be unreasonable for these data.

The analysis in Hayakawa *and others* (2003) assumes the same model as ours, but with infection rates constant through time. Posterior mean (standard deviation) infection rate estimates of 0.0045 (0.0018), 0.0018 (0.00082), and 0.0013 (0.00038) were obtained for infants, children, and adults, respectively, using MCMC methods. These are clearly comparable with our results.

### 5.4. London measles data

Our final example shows that the idea of using GPs as a prior distribution for an infection rate function can also be applied to other settings. We specifically consider an application to an inference method for large populations. The full details are rather extensive and can be found in Xu (2015); here we just report the key aspects.

**Fig. 3.**
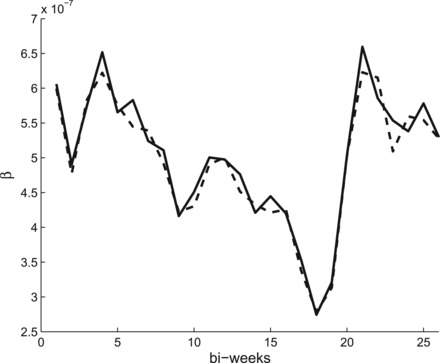
Posterior mean of the infection rates over 1 year for the CF method (dashed line) and NP method (solid line) using the squared exponential covariance function for the GP for the London measles data.

**Fig. 4.**
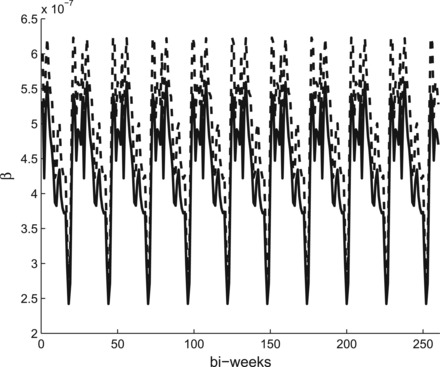
Posterior mean of the infection rates over 10 years for the CF method (dashed line) and NP method (solid line) using the periodic covariance function for the GP for the London measles data.

Consider data aggregated into time intervals (such as weeks or fortnights) consisting of numbers of new infections occurring during each time interval. For an SIR model, inference for the infection rate over any such time interval could in theory be achieved by imputing the numbers and times of infection and removal events, but in practice this approach breaks down in large populations unless the time interval is small, because the parameter space becomes too large to explore efficiently. Cauchemez and Ferguson (2008) tackle this problem by (i) assuming that the number of susceptibles in a time interval is constant, which implies that the infectives process is now a linear birth–death process; (ii) using an analytically tractable diffusion process to approximate the birth–death process, yielding a tractable approximation to the true likelihood. The methods also allow incorporation of data on new susceptibles, such as the number of births in each time interval in the context of childhood diseases, and estimation of the case reporting rate.

Cauchemez and Ferguson (2008) applied this method to fortnightly pre-vaccination-era data on measles in London, in which the data consist of numbers of new cases and births in each bi-weekly time interval. The authors estimated the infection rate for each time interval during a year, $\beta _0, \ldots , \beta _{25}$, say, under the assumption that the infection rates are viewed as independent parameters with no structural dependence. In reality we might expect to see some relationship between infection rates, e.g. $\beta _1$ might be close to $\beta _0$ and $\beta _2$. Cauchemez and Ferguson (2008) also modeled year-by-year trends by using the same infection rate parameters every year, but assuming that the incidence rate in each bi-weekly period was inversely proportional to the size of the number of children under the age of 4, itself known from the available data. In our framework, an alternative way to allow annual variation is to include a separate infection rate parameter for each 2-week time period in the entire data set, but then impose a GP prior on these parameters with a suitable periodic covariance structure.

We introduced these aspects by implementing the Cauchemez–Ferguson (CF) method with a non-parametrically modeled infection rate with a GP prior distribution. Specifically, we assume that the infection rate is constant in each fortnightly time interval, and then regard the mid-point of each interval as an input for the GP. Two scenarios are adopted: first, that the infection rate function was the same year-by-year, and second that it was not. We also implemented the CF method, without the trend modeling, for comparison. Cauchemez and Ferguson (2008) also considered incidence rates of the form $\beta X(t) Y(t)^{1 - \epsilon }$, which we do not consider here since our focus is toward assessing and illustrating our non-parametric approach, and extra model complexity reduces the transparency of these activities. We used 10 years of measles data, 1948–1957. Both our method and the CF method involve imputation of variables necessary to compute a likelihood, namely the number of unobserved cases in each time interval, the number of infectives at the start of each time interval, and the number of susceptibles at the start of the first time interval. Both methods also assume that the mean infectious period is 14 days. For our second non-parametric method, we used the periodic covariance function

\[ K(x,y) = \alpha^2 \exp (- \theta^{-1}(1 - \cos(2 \pi \omega^{-1} | x - y | ))), \]

in which $\omega $ represents the length of one period, which we set equal to 364 days (i.e. 26 bi-weeks, each 14 days). Rather than fixing $\alpha ,$ we included it as a model parameter, and assigned $\alpha $ and $\theta $ independent $U(0,10^7)$ prior distributions.

Figure 3 shows a comparison of the posterior mean of the infection rates $\beta _0, \ldots , \beta _{25}$ from the CF method and the Bayesian non-parametric method, the latter using a squared exponential covariance function for the GP prior. Both methods produce relatively similar estimates. Figure 4 compares the CF method with the periodic covariance function, again showing posterior mean values. Here, the CF method is as before, so each year has identical estimates, as shown in Figure 3. It appears visually that there is relatively little variation year-by-year in the non-parametric estimate; in fact, there are differences, but they are very small, typically of the order $10^{-11}$. We also attempted to use the CF method by individually estimating an infection rate for each of the 260 bi-week intervals, but the mixing of the MCMC algorithm was prohibitively poor. Our periodic covariance function method therefore appears to offer a viable alternative in this setting.

Table 1 shows some posterior parameter estimates for the three methods. Broadly speaking these are fairly similar, although there are some differences in the estimate of the initial number of susceptibles.

**Table 1.** Posterior mean, equal-tailed $95\%$ credible intervals, and posterior standard deviation for the reporting rate, $\rho ,$ the initial number of susceptibles $S_0$ and infectives $I_0,$ and two illustrative infection rates for the London measles data. CF, NP, and NP periodic represent, respectively, the CF method, the non-parametric method using the squared exponential covariance function for the GP prior with seasonal infection rates, and the non-parametric method using the periodic covariance function for the GP prior without assuming the infection rates are seasonal

	CF	NP	NP periodic
$\rho $$(\%)$	50.87 [50.69, 51.05] (0.11)	50.71 [50.56, 50.87] (0.10)	50.26 [50.09, 50.44] (0.11)
$S_0 \,(\times 10^3)$	164 [160, 168] (2.6)	161 [158, 164] (2.1)	185 [180, 190] (3.0)
$I_0$	603 [551, 659] (33.3)	605 [550, 661] (33.7)	602 [547, 658] (33.9)
$\beta \_0 \,(\times 10^{-7})$	5.99 [5.81, 6.17] (0.11)	6.08 [5.92, 6.24] (0.10)	5.31 [5.15, 5.47] (0.10)
$\beta \_10 \,(\times 10^{-7})$	4.91 [4.74, 5.06] (0.10)	5.02 [4.88, 5.17] (0.09)	4.18 [4.05, 4.32] (0.08)

## 6. Discussion

We have demonstrated that Bayesian non-parametric inference for epidemic models can be achieved using GP methods. Although our work is preliminary, it appears worthy of further exploration. The methods that we have developed can be extended to other settings, such as epidemic models with non-exponential infection periods and with latent periods, and those with more complex population mixing structures.

We have largely employed a squared exponential covariance function to define a prior for the GP of interest in our examples. However, we found that using other covariance functions such as exponential or Matern class functions did not have a material impact on the results, with the main difference in results being the smoothness of the posterior estimates for the infection rate functions. More details can be found in Xu (2015). A more general question is how best to choose a covariance function; this is partly an issue of model assessment, i.e. selecting a covariance function to give the best fit to data, and an interesting avenue for future research. In some settings certain covariance functions might arise naturally, as illustrated by our use of a periodic covariance function in the measles data example.

One practical challenge with our methods is that the computational complexity increases with the size of the problem. Specifically, calculating the multivariate Gaussian density functions involves inversion of the GP covariance matrix, an operation of typical complexity in the range ${\rm O}(n^{2.5})$ to ${\rm O}(n^3)$ for an $n \times n$ matrix, the size of which increases with the number of infections and thinned infection events. We found that the number of thinned events was often between two and three times larger than the number of infections. In practice we found that a data set with around 100 infections took around 1–2 h to run, increasing to 3–5 h for 200 infections. It is possible that recent developments in matrix inversion (e.g. Chen and Yi, 2016) could be fruitfully applied to reduce computation times. Nevertheless, as demonstrated in the measles example, our methods can be adapted via suitable approximating models.

## Funding

This work supported by the University of Nottingham (X.X.), the European Union's Seventh Framework Programme FP7-Health-2012-INNOVATION [grant agreement number 305280 (MIMOmics)] (X.X.), and the UK Engineering and Physical Sciences Research Council [grant number EP/J013528/1] (T.K.). Funding to pay the Open Access publication charges for this article was provided by Research Councils UK.

## Supplementary Material

Supplementary Data
