# Supplementary material for: Bayesian non-parametric inference for stochastic epidemic models using Gaussian Processes
Source: Biostatistics. 2016 Mar 18;17(4):619–33. doi: 10.1093/biostatistics/kxw011 (PMC5031942; doi:10.1093/biostatistics/kxw011)
Supplement: Supplementary Data [file kxw011_supp.pdf]

Biostatistics (2016), **0**, 0, *pp.* 1–13
doi:10.1093/biostatistics/Xu˙Kyp˙ON˙BNP˙supp

Bayesian nonparametric inference for stochastic epidemic models using Gaussian Processes: Supplementary Material

XIAOGUANG XU

Institute of Population Health, University of Manchester, Manchester

THEODORE KYPRAIOS, PHILIP D. O'NEILL*

School of Mathematical Sciences, University of Nottingham, Nottingham

philip.oneill@nottingham.ac.uk

Supplementary Material

We now describe the MCMC algorithms used in the paper, and also the results of a simulation study. The MCMC algorithms are similar to those described in O'Neill and Roberts (1999) and Hawakaya *et al.* (2003), with the additional feature of the Gaussian Process components. Here we provide brief details of the algorithms, both of which consist of individual updating steps for all the model parameters and imputed quantities, as described below. These steps are carried out sequentially. In practice, some of the steps may be repeated several times to improve the mixing of the Markov chain; this especially applies to updates for the infection times and thinned events. In the sequel, notation such as $\pi(\theta|\cdots)$ denotes the conditional density (or mass function, as appropriate) of θ given the data and all other parameters.

*To whom correspondence should be addressed.

© The Author 2016. Published by Oxford University Press. All rights reserved. For permissions, please e-mail: journals.permissions@oup.com

1. MCMC algorithm for the infection rate method

The examples in the paper assume that the epidemic is known to have finished by time T, and thus the number of infection events and removal events in group i is equal, i.e. $m_i = n_i$ for $i = 1, \dots, k$. If this is not the case then we can easily modify the algorithm below along the lines described in O'Neill and Roberts (1999), specifically allowing infection times updates that feature addition and deletion of infection times.

1. Sampling γ

We use a Gibbs step to sample from the full conditional density $\pi(\gamma|\cdots)$. With a gamma prior $\Gamma(\nu_\gamma, \lambda_\gamma)$ on γ, we have

$$\gamma|\cdots \sim \Gamma\left(\nu_\gamma + \sum_{i=1}^{k} n_i, \lambda_\gamma + \sum_{i=1}^{k} \int_{I_{i1}}^{T} Y_i(s)\mathrm{d}s\right).$$

2. Sampling $I_{\min}$

Recall that we scale time such that the first removal is at time zero, and that $I_{\min}$ has prior density given by $\eta \exp(\eta y)\mathcal{I}_{\{y<0\}}$, where $\eta > 0$, and $\mathcal{I}_A$ is the indicator function of the event A. Then if the first infection time is in group i we have

$$\pi(I_{\min}|\cdots) = \Lambda \exp\{-\Lambda(I_2 - I_{\min})\}, \quad y \in (-\infty, I_2),$$

where $I_2 = \min(I_{i2}, \{I_{j1}\}_{j=1, j\neq i}^{k})$ and $\Lambda = \eta + \gamma + N\tilde{\beta}_i^*$.

3. Sampling $\mathbf{I}$

An infection time I_{ij} is selected uniformly at random from those in $\mathbf{I}$, i.e. all infection times other than $I_{\min}$. A proposed new infection time I'_{ij} is drawn uniformly at random from the interval $[I_{\min}, T]$. Then a new value $g_i(I'_{ij})$ is sampled by conditioning on the existing value of $\mathbf{g}_{M_i+m_i}$, i.e. from a Gaussian distribution with mean $K(I'_{ij}, \mathbf{X})K(\mathbf{X},\mathbf{X})^{-1}\mathbf{g}_{M_i+m_i}$ and variance $K(I'_{ij}, I'_{ij}) - K(I'_{ij}, \mathbf{X})K(\mathbf{X}, \mathbf{I}_i)^{-1}(\mathbf{X}, I'_{ij})$, where $\mathbf{X} = (I_{i1}, I_{i2}, \dots, I_{im_i}, \tilde{I}_{i1}, \tilde{I}_{i2}, \dots, \tilde{I}_{iM_i})$. The acceptance ratio is

$$\frac{\sigma(g_i(I'_{ij}-))f(\mathbf{I}')}{\sigma(g_i(I_{ij}-))f(\mathbf{I})}$$

where $\mathbf{I}'$ denotes the proposed new value of $\mathbf{I}$ and

$$f(\mathbf{I}) = \prod_{p=1}^{k}\left(\left(\prod_{l=2}^{m_i} X_p(I_{pl}-)Y(I_{pl}-)\right)\left(\prod_{l=1}^{n_i} Y_p(R_{pl}-)\right)\right)\left(\prod_{p=1,p\neq i_{\min}}^{k} X_p(I_{p1}-)Y(I_{p1}-)\right)$$
$$\times \exp\left(-\sum_{p=1}^{k}\left(\int_{I_{\min}}^{T}\tilde{\beta}_i^* X_p(s)Y(s)\mathrm{d}s + \int_{I_{p1}}^{T}\gamma Y_p(s)\mathrm{d}s\right)\right).$$

4. Sampling $\tilde{\beta}_i^*$

If $\tilde{\beta}_i^*$ is assigned a $\Gamma(\nu_{\tilde{\beta}}, \lambda_{\tilde{\beta}})$ prior distribution then the full conditional density of $\tilde{\beta}_i^*$ is

$$\tilde{\beta}_i^* | \ldots \sim \Gamma\left(\nu_{\tilde{\beta}} + m_i - \delta_i + M_i, \lambda_{\tilde{\beta}} + \int_{I_{\min}}^{T} X_i(s)Y(s)\mathrm{d}s\right),$$

where $\delta_i = \mathcal{I}_{\{i=i_{\min}\}}$.

5. Sampling M_i

To update M_i, with equal probability either an insertion or deletion step is chosen. For the insertion step, a new thinned event is added and its location is chosen in an identical manner to that described above for proposed new infection times. For a deletion, one of the thinned events is chosen uniformly at random and deleted. The resulting acceptance ratios for insertion and deletion are, respectively,

$$\frac{(T - I_{i1}) \times \tilde{\beta}_i^* \times \sigma(-g_i(\tilde{I}'_{is}-)) \times X_i(\tilde{I}'_{is}-)Y(\tilde{I}'_{is}-)}{M_i + 1},$$

and

$$\frac{M}{(T - I_{i1}) \times \tilde{\beta}_i^* \times \sigma(-g_i(\tilde{I}_{is}-)) \times X_i(\tilde{I}_{is}-)Y(\tilde{I}_{is}-)},$$

where $\tilde{I}'_{is}$ and $\tilde{I}_{is}$ denote the proposed inserted and deleted thinned event times, respectively.

6. Sampling $\{\tilde{I}_{is}\}_{s=1}^{M_i}$

Updating of the thinned event times is performed in a similar manner to the infection time updates described above, i.e. a thinned event $\tilde{I}_{is}$ is chosen uniformly at random in group i and replaced with a proposed new thinned event $\tilde{I}'_{is}$ sampled uniformly on the interval $[I_{i1}, T]$. The

resulting acceptance ratio is

$$\frac{X_i(\tilde{I}'_{is}-)Y_i(\tilde{I}'_{is}-)\times\sigma(-g(\tilde{I}'_{is}-))}{X_i(\tilde{I}_{is}-)Y_i(\tilde{I}_{is}-)\times\sigma(-g(\tilde{I}_{is}-))}.$$

7. Sampling $\mathbf{g}_{M_i+m_i}$

Following Adams *and others* (2009), we propose new function values $\mathbf{g}'_{M_i+m_i}$ using a proposal of the form $\mathbf{g}'_{M_i+m_i} = \nu\mathbf{g}_{M_i+m_i} + \sqrt{1-\nu^2}\mathbf{h}_{M_i+m_i}$, where $\mathbf{h}_{M_i+m_i}$ is drawn from the Gaussian process at $\mathbf{I}$ and $\{\tilde{I}_s\}_{s=1}^M$ and $\nu\in[0,1)$. The acceptance ratio is

$$\frac{\prod_{i=1}^k\left(\prod_{j=1+\delta_i}^{m_i}\sigma(g'(I_{ij}-))\prod_{s=1+\delta_i}^{M_i}\sigma(-g'(\tilde{I}_{is}-))\right)}{\prod_{i=1}^k\left(\prod_{j=1+\delta_i}^{m_i}\sigma(g(I_{ij}-))\prod_{s=1+\delta_i}^{M_i}\sigma(-g(\tilde{I}_{is}-))\right)},$$

where $\delta_i = \mathcal{I}_{\{i=i_{\min}\}}$.

8. Sampling θ_i

Recall that we assign an exponential prior on θ_i, i.e. $\theta_i\sim\text{Exp}(\lambda_\theta)$. Then we propose a new θ'_i from a normal distribution with mean θ_i and variance, σ_i^2. The acceptance ratio for this proposal is

$$\frac{|\Sigma_{\theta'_i}|^{-\frac{1}{2}}\times\exp(-\mathbf{g}_{M_i+m_i}{}^T\Sigma_{\theta'_i}{}^{-1}\mathbf{g}_{M_i+m_i}/2-\lambda_\theta\theta'_i)}{|\Sigma_{\theta_i}|^{-\frac{1}{2}}\times\exp(-\mathbf{g}_{M_i+m_i}{}^T\Sigma_{\theta_i}{}^{-1}\mathbf{g}_{M_i+m_i}/2-\lambda_\theta\theta_i)},$$

where Σ_{θ_i} denotes the covariance matrix of $\mathbf{g}_{M_i+m_i}$.

2. MCMC algorithm for the incidence rate method

1. Sampling γ

We use a Gibbs step. With a conjugate gamma prior $\Gamma(\nu_\gamma,\lambda_\gamma)$ assigned to γ, we have

$$\gamma|\ldots\sim\Gamma\left(\nu_\gamma+K,\lambda_\gamma+\int_{I_1}^T Y(s)\mathrm{d}s\right).$$

2. Sampling I_1

We use a Gibbs step to sample I_1. Recall that I_1 has prior density given by $\eta\exp(\eta y)\mathcal{I}_{\{y<0\}}$, where $\eta>0$, and $\mathcal{I}_A$ denotes the indicator function of the event A. Then

$$\pi(y|\ldots)=\Lambda\exp\{-\Lambda(I_2-y)\},\quad y\in(-\infty,I_2),$$

where $\Lambda = \eta + \gamma + \beta^*$.

3. Sampling I

One of the current infection times is chosen uniformly at random and a proposed new time I_j' is sampled uniformly from $[I_1, T]$. Next we draw a new function value $\mathbf{g}(I_j'-)$ from the Gaussian distribution with density $\pi(\mathbf{g}(I_j'-)|I_j', \mathbf{g}_{M+K}, \mathbf{I}, \{\tilde{I}_s\}_{s=1}^M, \theta)$, with mean and variance $K(I_j', \mathbf{X})K(\mathbf{X}, \mathbf{X})^{-1}\mathbf{g}_{M+K}$ and $K(I_j', I_j') - K(I_j', \mathbf{X})K(\mathbf{X}, \mathbf{X})^{-1}(\mathbf{X}, I_j')$ respectively, where $\mathbf{X} = (I_2, I_3, \cdots, I_K, \tilde{I}_1, \tilde{I}_2, \cdots, \tilde{I}_M)$ denotes locations of infection times and thinned events. The acceptance ratio is

$$\chi' \times \frac{\sigma(g(I_j'-))}{\sigma(g(I_j-))} \times \frac{\prod_{i=1}^K Y'(R_i-) \exp\left\{-\int_{I_1}^T Y'(s)\mathrm{d}s\right\}}{\prod_{i=1}^K Y(R_i-) \exp\left\{-\int_{I_1}^T Y(s)\mathrm{d}s\right\}},$$

where $\chi' = 1$ if and only if there is at least one infective and one susceptible at each proposed infection time; otherwise, $\chi = 0$.

4. Sampling β^*

With a gamma prior $\Gamma(\nu_\beta, \lambda_\beta)$ assigned to β^*, we can update β^* using its full conditional distribution, $\Gamma(\nu_\beta + K + M - 1, \lambda_\beta + T - I_1)$.

5. Sampling M

To update M, with equal probability either an insertion or deletion step is chosen. For the insertion step, a new thinned event is added and its location is chosen in an identical manner to that described above for proposed new infection times. For a deletion, one of the thinned events is chosen uniformly at random and deleted. The resulting acceptance ratios for insertion and deletion are, respectively,

$$\frac{(T - I_1) \times \beta^* \times \sigma(-g(\tilde{I}_s'-))}{M+1},$$

and

$$\frac{M}{(T - I_1) \times \beta^* \times \sigma(-g(\tilde{I}_s-))},$$

where $\tilde{I}'_s$ and $\tilde{I}_s$ denote the proposed inserted and deleted thinned event times, respectively.

6. Sampling $\{\tilde{I}_s\}_{s=1}^M$

Updating of the thinned event times is performed in a similar manner to the infection time updates described above, i.e. a thinned event $\tilde{I}_s$ is chosen uniformly at random and replaced with a proposed new thinned event $\tilde{I}'_s$ sampled uniformly on the interval $[I_1, T]$. The resulting acceptance ratio is

$$\frac{\sigma(-g(\tilde{I}'_s-))}{\sigma(-g(\tilde{I}_s-))}.$$

7. Sampling $\mathbf{g}_{M+K}$

We propose new function values $\mathbf{g}'_{M+K}$ using a proposal of the form $\mathbf{g}'_{M+K} = \nu\mathbf{g}_{M+K} + \sqrt{1-\nu^2}\mathbf{h}_{M+K}$, where $\mathbf{h}_{M+K}$ is drawn from the Gaussian process at $\mathbf{I}$ and $\{\tilde{I}_s\}_{s=1}^M$ and $\nu \in [0, 1)$. The acceptance ratio is

$$\frac{\prod_{j=2}^K \sigma(g'(I_j-)) \prod_{s=1}^M \sigma(-g'(\tilde{I}_s-))}{\prod_{j=2}^K \sigma(g(I_j-)) \prod_{s=1}^M \sigma(-g(\tilde{I}_s-))}.$$

8. Sampling θ

Recall that we assign an exponential prior on θ, i.e. $\theta \sim \text{Exp}(\lambda_\theta)$. Then we propose a new θ' from a normal distribution with mean θ and variance σ^2. The acceptance ratio is

$$\frac{|\Sigma_{\theta'}|^{-\frac{1}{2}} \times \exp(-\mathbf{g}_{M+K}{}^T \Sigma_{\theta'}{}^{-1} \mathbf{g}_{M+K}/2 - \lambda_\theta \theta')}{|\Sigma_\theta|^{-\frac{1}{2}} \times \exp(-\mathbf{g}_{M+K}{}^T \Sigma_\theta{}^{-1} \mathbf{g}_{M+K}/2 - \lambda_\theta \theta)},$$

where Σ_θ denotes the covariance matrix of $\mathbf{g}_{M+K}$.

3. Simulation Study

We considered three scenarios; two single-type models and one multi-type model with three types of individual. The parameter values for each scenario are shown in Table 1. For each scenario we simulated 50 data sets, and then applied our methods to the resulting data sets. As mentioned in the main text, the methods can be time-consuming to run which is why we did not use more

Table 1. Parameter values used for three model scenarios (M1-M3). Parameters are infection rate $\beta(t)$, removal rate γ, initial number of susceptibles N, and initial number of infectives a.

	$\beta(t)$	γ	N	a
M1	0.015	1	150	1
M2	$\exp(-t/10)/150$	0.7	300	1
M3: group 1	0.005	0.5	25	0
M3: group 2	0.005	0.5	36	0
M3: group 3	0.002	0.5	192	1

simulated data sets. Nevertheless, the results appear sufficient to demonstrate that our methods work fairly well.

3.1 *Results from a single simulation*

Before presenting the full simulation results, we consider the results based on a typical single simulation from model M1, in order to illustrate what happens in practice. In the particular simulated data set we used, 130 of the initial susceptibles became infected.

Figure 1 shows posterior summaries from this data set for the infection and incidence rates. The former shows that the posterior mean is reasonably close to the true value (we would not necessarily expect it to be identical since the data are only from one possible outbreak) and in particular shows the infection rate does not vary appreciably throughout the epidemic. The plot for $\beta(t)$ also shows that the method appears to recover the truth reasonably well. The uncertainty around $\tilde{\beta}(t)$, shown via the credible interval width, increases towards the end of the outbreak, although for $\beta(t)$ the uncertainty decreases. An explanation for this is that although fewer observed cases suggests that the overall incidence rate is likely to be smaller, such data make it hard to separate out the contributions from the number of infectives and the infection rate itself - in other words, a precise estimate of $\beta(t)Y(t)$ does not yield a precise estimate of both components.

For comparison, Figure 1 also shows the mean value of $\tilde{\beta}(t)X(t)Y(t)$, which is an alternative

estimate of the incidence rate. It appears to perform slightly better than the direct estimate of $\beta(t)$, which is unsurprising since the method uses the additional assumption (which in this case is true) that the incidence rate is proportional to $X(t)Y(t)$. In practice we usually wish to estimate the incidence with as few assumptions as possible.

3.2 *Results from repeated simulations*

Figure 2 shows the mean estimates of the infection rates and incidence rates from 50 simulated data sets from model M1. All simulations were included, regardless of the number of infections that occurred. It can be seen that the true values are recovered fairly well. The mean credible intervals for the infection rate become larger from around $t = 4$ onwards, but this is almost certainly a reflection of the fact that as time increases, fewer of the simulated epidemics will have lasted that long. Specifically, among the 50 simulated data sets, around half had overall durations less than $t = 4$ time units.

Figure 3 shows that our methods can recover a non-constant infection rate function, specifically here inferring the fact that the true infection rate is decreasing over time. Again, the reduced accuracy towards the end of the time period is most likely attributable to having fewer epidemics of sufficient duration: more than half the simulated outbreaks died out rapidly. For the overall incidence, there is some underestimation around the peak of the epidemic, although the true mean incidence is still within the credible intervals. Again, this is likely to be at least partly attributable to the number of outbreaks that had sufficient duration. It is also possible that a different choice of kernel might improve estimation around the epidemic peak, although even our relatively simple kernel does a reasonable job overall.

Figure 4 shows that the method appears effective in the multi-type setting. The results also provide some evidence that the methods work well even for small outbreaks, since the average final numbers infected in the simulated data in groups 1, 2 and 3 were 5.3, 7.4 and 22.4 respectively, and

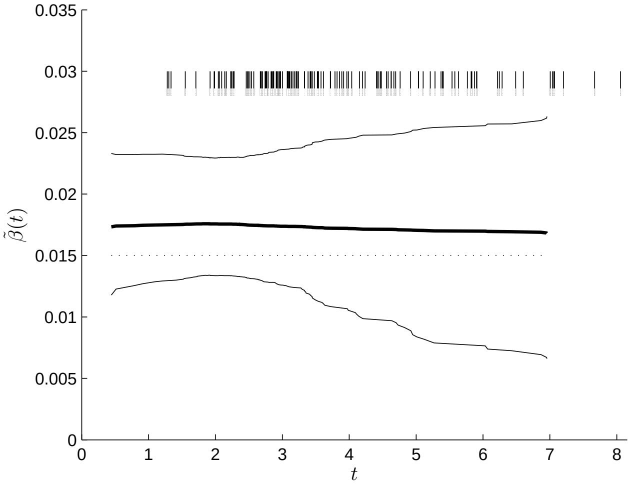

(a)

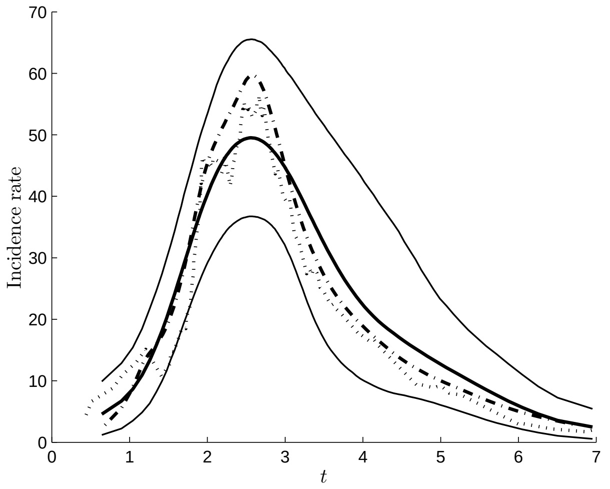

(b)

Fig. 1. Estimation of the infection rate (a) and incidence rate (b) for a single data set from model M1. Both plots show the true value (dotted line), posterior mean (thick line), 95% posterior credible intervals (thin line) and the upper plot shows the removal times in the data set (vertical dashes at top of plot). The lower plot also shows mean incidence estimated as $\tilde{\beta}(t)X(t)Y(t)$ (dashed-and-dotted line). All curves are plotted over the mean posterior time during which infectives were present in the population.

around 40% of the outbreaks in the data had no infected individuals at all (it is straightforward to show that the probability of the epidemic dying out immediately is around 0.42). The fact that we have relatively few data points also explains why the plots are not particularly smooth.

Finally, we expect the results to improve further with more simulations. We initially used 30 simulated data sets for each scenario, and found a discernible improvement in mean estimation performance when we increased the number to 50. As mentioned previously, the time taken to perform such analyses is one prohibitive feature of our methods, and it would be of benefit to develop methods for reducing the computation times involved.

References

Adams, R. P., Murray, I., and MacKay, D. J. C. (2009). The Gaussian process density sampler. *Advances in Neural Information Processing Systems* **21**.

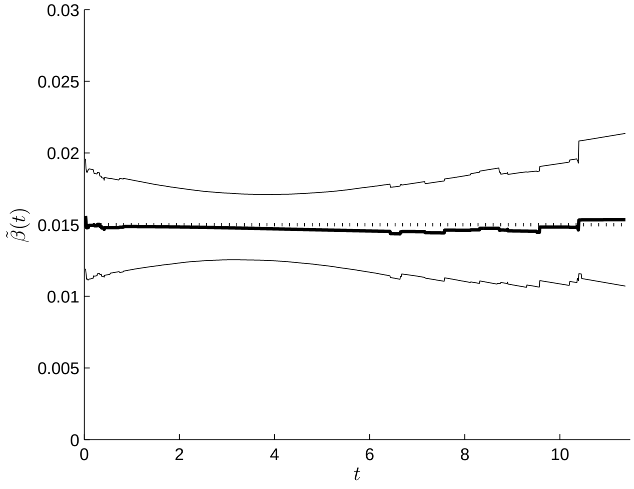

(a)

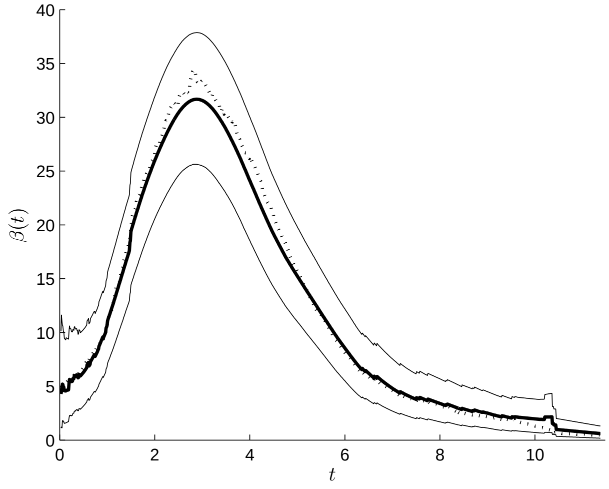

(b)

Fig. 2. Estimation of the infection rate (a) and incidence rate (b) from 50 simulated data sets from model M1. Both plots show the mean true value (dotted line), mean posterior mean (thick line), and mean 95% posterior credible intervals (thin line).

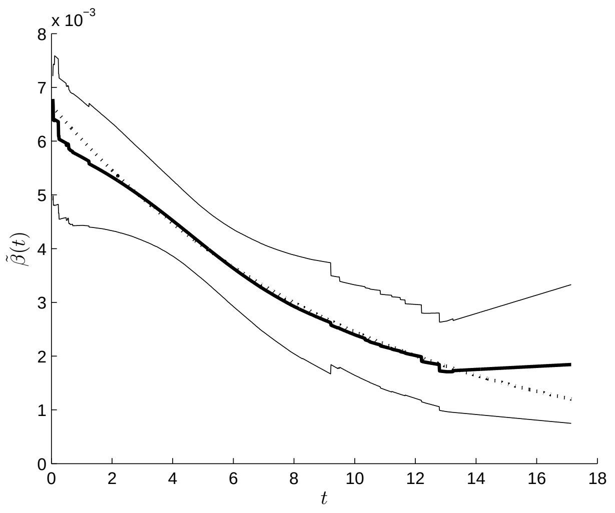

(a)

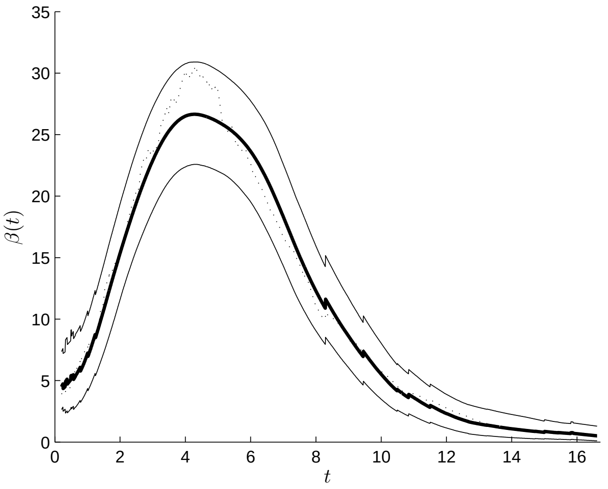

(b)

Fig. 3. Estimation of the infection rate (a) and incidence rate (b) from 50 simulated data sets from model M2. Both plots show the mean true value (dotted line), mean posterior mean (thick line), and mean 95% posterior credible intervals (thin line).

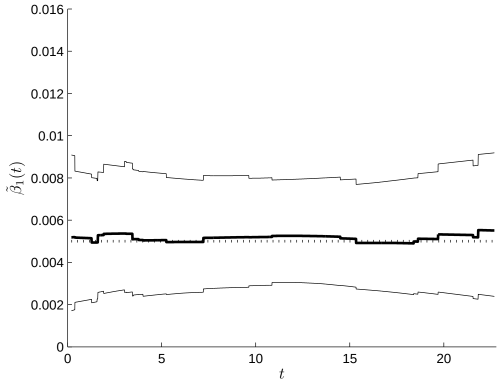

(a)

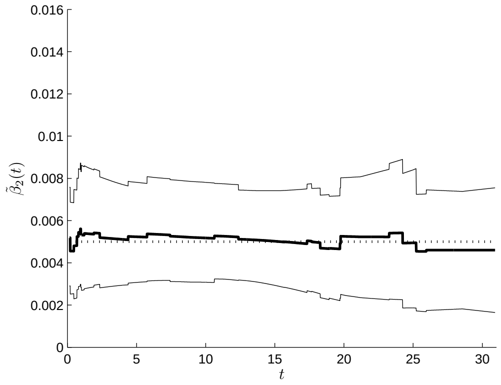

(b)

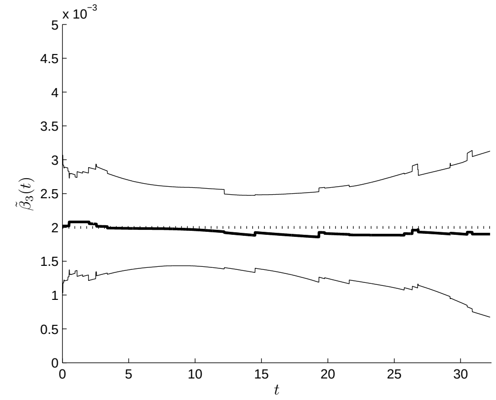

(c)

Fig. 4. Estimation of the infection rate from 50 simulated data sets from model M3. All plots show the mean true value (dotted line), mean posterior mean (thick line), and mean 95% posterior credible intervals (thin line).
